# Remote Interventions to Support Students’ Psychological Well-Being during the COVID-19 Pandemic: A Narrative Review of Recent Approaches

**DOI:** 10.3390/ijerph192114040

**Published:** 2022-10-28

**Authors:** Anna Rutkowska

**Affiliations:** Department of Physical Education and Physiotherapy, Opole University of Technology, 45-758 Opole, Poland; a.rutkowska@po.edu.pl

**Keywords:** mental health, mental support, psychological support, COVID-19, students

## Abstract

The COVID-19 pandemic has negatively affected students’ mental health, and it is important to implement mental health management strategies. The purpose of this study was to present current findings on the implementation of remote mental health interventions in students during the pandemic. The PubMed and Web of Science electronic databases were searched and, from a total of 174 articles, 106 records were excluded according to the inclusion criteria and 23 were assessed as full texts. After the full-text screening, 12 studies were included in the review. The included publications were randomized clinical trials focused on remote mental support interventions among students from 10 countries, representing both genders, and were in the average age range of 17–55 years with an overall number of 892 participants. The included studies covered the effectiveness of strictly psychotherapeutic programs, such as cognitive-behavioral therapy (CBT) and dialectical behavior therapy (DBT), as well as other techniques such as mindfulness, laughter therapy, the brain wave modulation technique (BWM-T), and physical activity-based interventions. This narrative review provides an overview of studies with a wide range of types of remote mental health support interventions. Each of the forms of intervention analyzed in this review resulted in positive changes in students’ mental health, which indicates hope for widespread help via various forms of intervention implemented remotely.

## 1. Introduction

In March 2020, the World Health Organization declared the 2019 coronavirus disease (COVID-19) to be a pandemic [1]. From that date, the life of every citizen in the world changed as a result of the enforced restrictions and quarantine. The pandemic’s consequences on patients’ health status, economic and social effects, and the state of mental well-being of the population have been described. It is estimated that the prevalence of mental illness has increased by up to 25% since the pandemic outbreak [2]. The pandemic disrupted the sense of social security and strong emotional reactions appeared, causing feelings such as anger, helplessness, anxiety, fear, nervousness, irritability, frustration, stress, insomnia, depression, self-harm, post-traumatic stress disorder, and suicidal behavior [3,4,5,6]. These alterations are also noted in students who, even before the pandemic, were identified as being at risk for poor mental health relative to other groups [7,8]. The good mental health of students is a guarantor of academic success: it improves concentration, motivation to learn, and increases social interaction [9]. The long-term effects of the pandemic on students’ mental health are unknown but numerous studies report increased stress levels, depression, anxiety, insomnia, eating disorders, and suicidal thoughts and behaviors [10,11,12,13,14,15,16]. The prevalence of depression among students before the pandemic had settled at 30% but [8], based on the results from studies conducted in many countries, a significant increase in depressive symptoms can currently be observed: Spain, 31.19% (*n* = 2530); Lebanon, 33.4% (*n* = 520); Slovakia, 47% (*n* = 3051); the USA, 48.14% (*n* = 2031); China, 48.3 (*n* = 4872); France, 49.5% (*n* = 69,054); Poland, 56.4% (*n* = 753); and Greece, 60.9% (*n* = 1000) [11,12,13,14,15,16,17]. Scientists highlight that the strongest predictor of depression is elevated stress [18,19], while depression increases the risk of suicidal thoughts and suicide [20,21]. Moreover, e-learning has been shown to be a predictor of increased levels of stress and depression among students due to demonstrated limited social contact, decreased motivation to learn, negative impact on knowledge and fear of lower grades [16]. Time spent in front of a computer or phone screen has increased significantly as a result of distance learning and contact with colleagues through communicators, which predict a higher risk of anxiety and depression [22]. Furthermore, a reduced level of students’ physical activity was noted during the pandemic [23,24], as well as the negative impact of staying online for an excessive amount of time [25].

These circumstances indicate a high-risk for young adult functioning in a very important stage of life. This period is characterized by the search for one’s own identity and building life goals related to work, family, or social roles. However, this essential period has been altered for many millions of individuals by an unprecedented disruption of bio-psycho-social life. It seems crucial to recognize indicators that contribute to advances in understanding mental health problems and predictors of well-being in the emerging adult population. Thus, there is an urgent need for research to address how the mental health consequences among students can be mitigated under pandemic conditions. The purpose of this study was to present current findings on the implementation of remote mental health interventions and to verify their effectiveness in emerging mental health disorders among students during the pandemic.

## 2. Materials and Methods

### Electronic Searches

**Figure 1:**
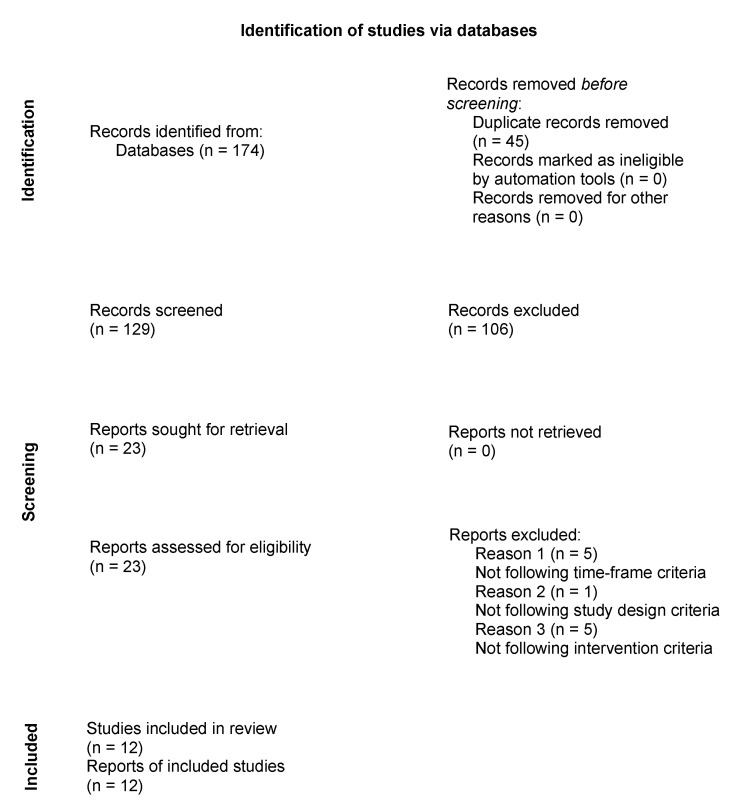
Flow diagram for the study selection process.

The literature search was conducted on 6 August 2022 using the PubMed database (National Library of Medicine, 8600 Rockville Pike, Bethesda, MD 20894, USA) and Web of Science (Clarivate, 1500 Spring Garden, Philadelphia, PA 19130, USA). To identify studies from a specific research topic, the COVID-19 filters from PubMed Clinical Queries were implemented. Due to the limited amount of research available, no specific publication dates were determined. The following search formula was defined for the PubMed database: “((((mental health OR anxiety OR stress OR depression) AND (students OR university OR college) AND (COVID-19 OR pandemic)) AND (psychological Intervention OR mobile mental health OR positive psychology OR mental support OR mindfulness OR meditation OR digital health OR chatbots OR CBT OR ICBT)) AND (Therapy/Broad [filter]). For the Web of Science database, the particular searches for the category and topic were done separately, the categories were combined OR, while the individual topic search phrases were combined AND. The following formulations were used: WC = (1) Telecommunications; (2) (Psychology, Multidisciplinary); TS = (1) (“psychological Intervention” OR “mobile mental health” OR “positive psychology” OR “mental support” OR mindfulness OR meditation OR “digital health” OR chatbots OR CBT OR ICBT); (2) (COVID-19 OR pandemic); (3) (students OR university OR college); (4) (mental health OR anxiety OR stress OR depression). The electronic search identified 174 overall results, with no additional records from the gray literature search. In this review, it was decided to include only randomized controlled trials (RCTs) aimed at interventions to counteract the effects of a COVID-19 pandemic on the student group in order to present a wide range of interventions. After removing 45 duplicates, 129 abstracts remained for screening; 106 records were excluded due to their unrelated topic or wrong study design and 23 full-text articles were analyzed. After the full-text screening, 12 studies met the inclusion criteria. The following inclusion criteria were implemented: (1) population: university students; (2) intervention: remote mental health support methods; (3) timeframe: studies undertaken after the COVID-19 pandemic was identified. The review process is presented in a PRISMA flowchart (Figure 1) [26].

## 3. Results

### 3.1. Characteristics of the Included Studies

The included publications were RCTs targeting interventions that affect students’ mental health during the COVID-19 pandemic. In all studies, participants represented both genders and were in the average age range of 17–55 years. The procedure included 12 studies from 10 countries. The overall number of participants in the included studies was 892 (Table 1).

### 3.2. Mindfulness Intervention Programmes

Simonsson et al. evaluated the effects of an eight-week online mindfulness program during the pandemic on anxiety and depression among college students. Students were randomly assigned to a study group (*n* = 88) and a control group (*n* = 89). The study group participated in the program, which consisted of eight weekly sessions and was conducted online via the Zoom platform by a mindfulness teacher. The session lasted 90 min and involved mindfulness meditation practices, periods of inquiry and reflection and interactive exercises based on cognitive-behavioral therapy (CBT). Participants were encouraged to engage with the home practice for 20–30 min per day. The Patient-Reported Outcomes Measurement Information System (PROMIS) scale was used to assess anxiety and depressive symptoms. Participants in the study group showed a significantly greater reduction in anxiety after participating in the mindfulness program compared to the control group. No changes were noted in the area of depressive symptoms between the groups [27].

A study by Celia et al. aimed to investigate the effects of online counseling sessions based on a mind-body technique (BWM-T). Authors incorporate the brain wave modulation technique for reducing negative affect and anxiety. The study included a total of 79 students: 54 assigned to the intervention group and 25 in the control group. The techniques sought to reduce the frequency of brain waves, which characterizes the state of relaxation and sleep. It involves a simple four-step finger movement, with the goal of activating the parasympathetic system. During the first session, a clinical psychologist demonstrated each of the four BWM-T finger positions. The study evaluated the effects of the intervention on depression levels as measured by the State-Trait Anxiety Inventory (STAI-Y) and positive and negative affects by the Positive and Negative Affect Schedule (PANAS). Participants in the online intervention group reported an increase in positive affect and a decrease in negative affect, as well as a modest decrease in anxiety compared to control group participants [28].

The study by Boyd et al. aimed to evaluate whether a six-week mindfulness-based intervention (MBI) in comparison with supportive counseling (SC) can increase resilience to stress in students. A total of 38 students (age range 19–30 years; 71% women) completed the programs: MBI and SC. The MBI program was implemented through the Zoom platform and included practices targeting the body, breath, thoughts, habits and external/internal experiences. The session lasted 60 min. The second SC program used common psychoeducation methods, including techniques aimed at stress reduction: stress wheel, awareness triangle exercises, role of emotions in stressful situations, muscle relaxation, and identification of goals and values. The study measured levels of perceived stress, well-being state, and levels of self-compassion. The results revealed significant improvement in both groups regarding well-being and perceived and managed stress. Participants in the MBI group showed a statistically significant treatment effect in mindfulness at program completion. Moreover, a decrease in self-compassion over time was observed in both groups. The authors conclude that both the MBI and SC programs are feasible and show potential for reducing stress, increasing stress management, and increasing resilience in students [30].

Devillers-Réolon et al. evaluated the efficiency of an online mindfulness meditation intervention in countering psychological distress and improving attentional abilities. Students were randomly assigned to an intervention group (*n* = 48) and a control group (*n* = 48). The mindfulness meditation (MM) program was introduced to the intervention group. The program included an introductory first online MM session and on subsequent days students were using an audio record made available on the University’s digital platform website. The online mindfulness intervention took 17 days (10–20 min per day). Result analyses showed a reduction in stress, anxiety and depression, and an improvement of well-being in the experimental group but not in the control group. In both groups, no significant effect was found on attentional abilities. The results of the study constitute further evidence of the feasibility and effectiveness of mindfulness-based interventions toward supporting students in coping with pandemic-induced psychological distress [31].

Sun et al. evaluated the effect of a mindfulness-based mobile health (mHealth) intervention among young adult students with elevated anxiety and/or depressive symptoms compared to a time- and attention-matched social support-based mHealth control. Students were randomly assigned to: mindfulness-based mHealth (*n* = 57) or social support-based mHealth (*n* = 57). The primary outcomes measured were the Patient Health Questionnaire 9 (PHQ-9) and Generalized Anxiety Disorder 7 (GAD-7) scales, while the Mindful Attention Awareness Scale (MAAS) and Multidimensional Scale of Perceived Social Support (MSPSS) were considered secondary outcomes. The intervention was based on a four-week “Mindfulness for Growth and Resilience” program and included weekly one-hour video conferences via Zoom and a WeChat-based mini-program that included 20 videos and audio recordings for daily audio-based mindfulness learning. Social support-based mHealth was delivered via Zoom and WeChat as four weekly one-hour sessions to discuss shared experiences and promote peer support. Analysis of the results showed that mindfulness-based mHealth has a superior effect on reducing anxiety, although both interventions had a similar effect on improving depressive symptoms. The authors concluded that a potential combination of self-learning, practice and a group component could significantly reduce the workload [32].

Fassnacht et al. aimed to evaluate whether the Be Well Plan online program is effective for improving mental well-being, resilience, anxiety, and depression among students. An RCT was conducted with an intervention group (*n* = 75) and a passive control group (*n* = 89). Participants in the intervention group took part in the Be Well Plan program, which is a weekly five-session online group intervention delivered online via Zoom by trained facilitators. The program instructs individuals to create a personalized mental health and well-being plan and focuses on self-exploration to determine key motivators, resources and challenges. The Be Well Plan program was effective in improving mental well-being, resilience, depression and anxiety. Participant satisfaction scores and attendance indicated a high degree of engagement and satisfaction with the program [36].

A study by Murray et al. compared the effects of an online exercise intervention (WeActive) and an online mindfulness yoga intervention (WeMindful) on anxiety and depression in students’ community during a pandemic. An RCT was conducted with the WeActive intervention (*n* = 46) and the WeMindful intervention (*n* = 29). The interventions took place twice a week for 30 min over a period of eight weeks by qualified instructors via the Zoom platform. In the WeActive group, students performed aerobic, resistance, and stretching exercises with increasing intensity. In the WeMindful group, the activity element was yoga and its components: breathing exercises, a sequence of movements and postures, and relaxation. Attendees in both groups had a significant decrease in depression scores, and no statistically significant decrease in anxiety symptoms. No significant changes were noted between the groups. The results of the study showed the effectiveness of both intervention programs for improving students’ mental health [37].

### 3.3. Behavioral Therapy

The purpose of the Rackoff et al. study was to evaluate the effectiveness of online self-help interventions in students with moderate or higher stress during a pandemic. The sample included 585 students. Participants were randomly assigned to online self-help (*n* = 301) or referral for usual care (*n* = 284). The intervention integrated principles of CBT and positive psychology. Students in the intervention group had access to two programs on SilverCloud Health. The program, “Space for Resilience,” used the principles of positive psychology to promote mental health in the face of stress, while the program, “Space from COVID-19” was aimed at promoting a sense of security, calm, coherence, and hope. Both programs were based on CBT and grief therapy techniques. Participants could use the two programs in any order and at any pace. The control group was provided with referral information on opportunities and places to implement mental support. The stress level was assessed using the Depression Anxiety Stress Scales (DASS). The results indicate a positive impact of the online intervention on reductions in stress and depression. Long-term effects of the intervention were also noted, following a re-evaluation after three months in the intervention group. The authors conclude that widespread access to online self-help services based on the principles of CBT and positive psychology may contribute to a reduction in the prevalence of mental health symptoms in students [29].

The study by Rizvi et al. purposed to evaluate the feasibility, acceptability and preliminary efficacy of short videos of dialectical behavior therapy (DBT) to reduce psychological distress among university students during the COVID-19 pandemic. Students were randomly assigned to an intervention group (*n* = 99) and a control group (*n* = 54). During the intervention phase, the study group received one video daily at 8 pm via the smartphone app for 14 consecutive days. An expert in DBT created narrated animated videos describing 14 DBT skills: distract, self-soothe, tip, improve the moment, radical acceptance, mindfulness “what” skills, wise mind, mindfulness “how” skills, opposite action, please, mindfulness of current emotion, give and fast, Dearman. The results obtained indicate that this type of intervention is feasible and acceptable. The authors concluded that DBT skills videos have the potential to support students to prevent mental health degradation [33].

Hanani et al. evaluated the effect of a CBT program on mental health among medical students during the COVID-19 pandemic. In a randomized procedure, 34 students were included in the intervention (CBT) group and 32 in the control group. Each 60-min weekly online session was via the Zoom platform (consisting of a lecture, discussion and training). The topics of the CBT program were: introducing program therapy; psycho-education; introducing negative thinking and thinking exposure; negative thinking becomes positive; relaxation and facing the anxiety situation; end of program and post-assessment. The control group was just provided with general information about mental health via WhatsApp messages. The CBT intervention program showed a significant improvement in depression, anxiety, and social dysfunction for the study group compared to the control [35].

### 3.4. Laughter Therapy

Eraydin et al. evaluated the effect of laughter therapy on anxiety, satisfaction with life, and psychological well-being among students during the COVID-19 pandemic. The intervention group consisted of 39 students and the control group comprised 40 students. Laughter therapy was applied to the intervention group for five weeks, two sessions a week, for ten sessions through the Zoom platform. Each laughter therapy session consisted of four parts. The first part includes hand clapping and warm-up exercises. In the second part, deep breathing exercises were implemented. The third part involves children’s games to induce and activate simulated laughter. In the fourth part, laughter exercises were performed. The applied intervention improved life satisfaction and well-being and reduced anxiety in the study group. The authors point out that laughter therapy is a safe, low-cost and cost-effective therapy that can be applied online to a greater range of students [34].

### 3.5. Physical Activity-Based Interventions

A study by Philippot et al. assessed the feasibility of a web-based high-intensity interval training (HIIT) program in students during the COVID-19 pandemic lockdown on psychological symptoms. Students were randomly assigned to an intervention group (*n* = 13) and a control group (*n* = 15). The intervention consisted of 12 intensive sessions of intermittent aerobic and muscle-strengthening exercises (burpees, mountain climbers, jumping jacks, squats, and jump forward lunges) with a perceived exertion index (RPE) of 6 or greater (max. 10) corresponding to a maximum heart rate of at least 80%. The recovery intervals included planks, lateral planks, push-ups, and squats at a requested RPE of 4 or less. For four weeks, participants received a block of three sessions, for a total of 12 sessions. Each 10-min session comprised alternating HIIT and active recovery intervals, each lasting 30 s. The DASS-21 was used to assess symptoms of depression, anxiety and stress. Analysis of the results revealed that HIIT reduced both stress and depressive symptoms in the study group. The results suggest that this type of intervention has good tolerance (at a participation rate of 87%) and has the potential to counter mental problems among young people [38].

## 4. Discussion

The COVID-19 pandemic has negatively impacted mental health among college students. This review presents a wide range and variety of interventions to improve mental health in this group. The content of these intervention programs varied widely. The authors evaluated the effectiveness of strictly psychotherapeutic programs such as CBT and DBT, as well as other techniques such as mindfulness, laughter therapy, BWM-T or physical activity-based interventions, each showing benefit for mental health improvement. In the included studies, anxiety and depressive symptoms were assessed most frequently. All the included studies followed a pretest-posttest design. Moreover, four were supplemented by an additional follow-up examination [27,29,30,35]. An analysis of the statistical methods used revealed that three authors used univariate pre-post methods (Wilcoxon test, t-test, Mann-Whitney U test) [34,35,38], six authors used linear models [27,29,32,33,36,37], three studies employed analysis of variance [28,30,31].

It is important to note that the interventions took a remote form. Web-based interventions have the advantage of being available regardless of location, which is especially important when access to face-to-face interventions is limited due to enforced restrictions or quarantine as a result of the current pandemic. It seems that the advantages of remote programs include the potential to cover large populations, and they constitute the first line of defense against mental health problems; above all, they can be an effective way of reducing stress and preventing the development of mental health conditions. The included studies showed that online interventions were rated as safe and attractive, with high feasibility and participation rates. Online interventions can also counteract some of the systemic inequalities that exist because they facilitate access in rural areas. They also provide a solution for people who are not ready or able to access medical services due to various barriers, such as cost or time, which can occur when students are assigned to remote learning.

Attention should be paid to the continued fortunes of students, their participation in the labor market and the development of societies. This social group holds the potential to indicate future development in particular areas of life, such as the humanities, life sciences, and technology. The lives of each of us in the coming decades depend on those individuals who are preparing to play a conscious role in society. This should be understood as participation in the labor market—guiding directions and revealing undiscovered resources or even opportunities to counter, as yet, unknown health or geopolitical dangers. It would seem to be the responsibility of current decision-makers to consider how the mental state of students may determine their opportunities in the future. It is not even necessary to look for specialized studies, considering the widely available knowledge in the field of mental health, in order to conclude that their mental well-being will affect their potential—and this will be seen in 10 or maybe 20 years. This very message is also the conclusion of this review: an attempt to motivate university management and future employers to pay attention to potentially fairly simple and accessible ways of supporting mental health that can be implemented as mandatory elements of education or duties. Familiarizing students with methods, techniques or support therapies can contribute to their ability to combat stress when they begin their careers.

Another area of the results of this review lies in the gender variability of symptoms. Almost 10 times more women are prone to stress compared to men [39], thus it may seem relevant that the included studies cover a larger sample of women. It has been reported that a number of industries most affected by the COVID-19 health crisis employ predominantly women, who are consequently at greater risk of losing their jobs and income [40,41]. Furthermore, women tend to be informal caregivers in families, which in practice often implies that the closure of schools and childcare facilities increases their workload at home [42]. This can significantly limit women’s ability to carry out their professional responsibilities, whether they work at home or on-site [43]. Moreover, gender differences in the prevalence of depression are widely reported, with women almost twice as likely as men to develop depression [44]. This leads to women having more difficulty keeping their jobs or being under greater psychological strain, which the review found can be offset by appropriate remote support methods. Therefore, it seems appropriate to develop different programs for women and men, as the studies revealed a higher association in the relationship between job stress and negative mental well-being among women than men [45,46].

Due to the great prevalence of mental health disorders among students, it is important to introduce support programs on a large scale. Among the methods presented, the proposed solution by Eraydin and colleagues seems interesting [34]. The intervention was based on laughter therapy, which has been shown to have benefits for the immune, cardiovascular, nervous, and endocrine systems since the mid-20th century [47]. Mental health disorders are characterized by changes in the production of serotonin and dopamine, which is often the cause of depression. Laughter therapy from a physiological perspective stimulates the production of these neurotransmitters [48]. The period of early adulthood and university studies should be associated with joy, smiling and fascination with life. It seems that laughter and humor is an integral part of a human lifetime, thus it is so important to enhance these positive emotions. The presented program could be used on a large scale, in an easily accessible online version at the low cost of implementation among students, bringing benefits in terms of improving mental health.

The authors of the included studies pointed out the limitations of the remote interventions that were applied. For the most part, the short-term effects of mental health support programs were examined. A longitudinal investigation is required to observe how long the benefit of the applied intervention persists and whether follow-up sessions would be an effective solution to boost it. This would increase statistical power, as well as our understanding of the effectiveness of the intervention in preventing the recurrence of psychiatric symptoms. Another mentioned limitation is the lack of objective monitoring of students during the implementation of the testing and intervention sessions. Participants were given detailed instructions and reminded during testing and sessions, and we cannot be sure how reliable and punctual they actually were. Ideally, for future investigations, pre-testing and post-testing should be conducted under laboratory-controlled conditions in the presence of an experimenter or specialist; a doctor or psychologist, who will further explain any doubts that may arise. Thus, the evaluation will be more reliable. The period of the pandemic and the restrictions that have been placed have probably limited researchers from such an opportunity. As with most investigations of psychological treatments, all outcome measurements were based on subjective evaluations of study participants. Future investigations could conduct evaluations using behavioral or physiological measures. For example, the use of technology could now facilitate evaluation using objective measures such as activity levels, sleeping patterns, or heart rate. Knowledge of whether particular remote interventions improve such outcomes would increase its utility as a prevention and early intervention program, representing a major relief of the health care service. In the included studies, there was a reduction in the number of participants who completed the investigation relative to the number recruited. The lack of continuous monitoring and communication with the experimenter made it impossible to collect information on the reasons for dropout from the investigation. Future investigations should promote higher levels of engagement and intrinsic motivation from participants and communication with other participants and investigators to encourage participants to complete the intervention and recommended tasks, thus reducing dropout rates. Another weakness is the technical requirements of hardware, adequate access to strong internet and skills in using computer equipment. The lack of these prevents or makes it difficult to participate in the intervention. However, it seems that in the student group these difficulties will occur less frequently than in the older group. In accordance with scientific reports on appearing differences in cultural competence in the medical professions practiced, it also seems relevant here to pay attention to raising skills in this area in expert groups that implement remote forms of mental health support [49]. This could be an important direction in personalizing the interventions used and increasing participation

## 5. Conclusions

The fact that each of the forms of intervention used in this review resulted in positive changes to students’ mental health is a positive conclusion. Students will be able to choose the form of intervention that attracts their interest most, depending on their preferences, which will result in attendance, engagement and a degree of positive impact. The hope for widespread help may consist of various interventions implemented remotely, and these should become more accessible. It seems that mobilizing populations to introduce mental support interventions now will enable the acquired knowledge to be applied in any future periods of pandemic and enforced restrictions. Reflecting on the results of this review, it should be noted that the effectiveness of remote therapies among the included studies were reported on small study samples, thus it should be considered as a good practice rather than a meta-analysis of the available literature.

## Figures and Tables

**Table 1:** Characteristic of included studies.

Authors, Year	Study Location	Participants	Intervention	Frequency	Assessment
Simonsson (2021) et al. [27].	United Kingdom	IG: 88 students	Mindfulness course	8-weekly on-line sessions	PROMIS
		Female 64.8%			
		Male 29.6%			
		Other 5.5%			
		CG: 89 students			
		Female 64%			
		Male 33.7%			
		Other 2.2%			
Celia (2021) et al. [28]	Italy	IG: 54 students	BWM-T	4-once-weekly online counselling sessions	PANAS STAI-Y
		CG: 25 students			
		Female: 96.3%			
		Male: 3.7%			
Rackoff (2022) et al. [29]	United States	IG: 301 students	CBT and positive psychology	Participants could use the two programs and their modules in any order and at any pace during one month from SilverCloud Health.	DASS
		Female 71.76%			
		Male 21.56%			
		Other 6.64%			
		CG: 284 students			
		Female 76.41%			
		Male 18.31%			
		Other 5.28%			
Boyd (2022) et al. [30]	Republic of South Africa	Total in both group 38 students	MBI	6 week on-line programme	CORE-OM WEMWBS PSS SCS-sf
		Female: 71.1%			
Devillers-Réolon (2022) et al. [31]	France	IG: 48 students	Online Mindfulness Intervention	17 days, (10–20 min per day).	DASS21 WEMWBS D2-R
		Female 47.9%			
		CG: 48 students			
		Female 25%			
Sun (2022) et al. [32]	China	IG: 57 students	Mindfulness-based mHealth	4-week on-line program	PHQ-9 GAD-7
		CG: 57 students			
		73% Female			
		27% Male			
Rizvi (2022) et al. [33]	United States	IG: 99 89students	DBT	14-days on-line intervention	Self-Efficacy for Managing Emotions—Short Form
		Female 47.9%			
		CG: 54 students			
		Female 92.59%			
Eraydin (2022) et al. [34]	Turkey	IG: 39 students	Laughter therapy	five weeks, two sessions a week, on-line	STAI SWLS PWBS
		Female 69.2%			
		CG: 40 students			
		Female 55%			
Hanani (2022) et al. [35]	Palestine	IG: 34 students	CBT	8 weeks; 60-min weekly on-line session	GHQ-12
		CG: 32 students			
Fassnacht (2022) et al. [36]	Australia	IG: 75 (49) students	Be Well Plan	5 weeks, once a week 2 h session	PHQ-9 GAD-7 CD-RISC-10 CFS WEMWBS NGSES SCS
		Female 81.3%			
		Male 17.3%			
		Other 1.3%			
		CG: 89 (54) students			
		Female 80.9%			
		Male 18%			
		Other 1.1%			
Murray (2022) et al. [37]	United States	IG 1: 46 students	IG1: WeActive IG2: WeMindful	8 weeks, twice a week, 30 min on-line session	GAD-7 MDI
		IG 2: 29 students			
		Female 85.1%			
		Male 9.5%			
		Other 5.3%			
Philippot (2022) et al. [38]	Belgium	IG: 13 students	HIIT	4-week program with three sessions per week	DASS-21
		Female 84.61%			
		Male 15.39%			
		CG: 15 students			
		Female 93.33%			
		Male 6.67%			

BWM-T = Brain Wave Modulation Technique; CBT = Cognitive Behavioral Therapy; DBT = Dialectical Behavior Therapy; CD-RISC = Connor-Davidson Resilience Scale; CFS = Cognitive Flexibility Scale; CORE-OM = Clinical Outcomes in Routine Evaluation—Outcome Measure; DASS = Depression, Anxiety, Stress Scale; GAD = General Anxiety Disorders; HIIT = High-Intensity Interval Training; PHQ = Patient Health Questionnaire; NGSES = New General Self-Efficacy Scale; MDI = Major Depression Inventory; MBI = Mindfulness-Based Interventions; PANAS = Positive and Negative Affect Schedule; PROMIS = Patient-Reported Outcomes Measurement Information System; PWBS = Psychological Well-Being Scale; PSS = Perceived Stress Scale; SCS = Sense of Control Scale; SCS-sf = Self-Compassion Scale—short form; STAI = State-Trait Anxiety Inventory; SWLS = The Satisfaction with Life Scale; WEMWBS = Warwick-Edinburgh Mental Well-being Scale.

## Data Availability

Not applicable.
